# Surgical Repair of a Quadricuspid Aortic Valve With Severe Regurgitation Utilizing “Tricuspidization” and Annular Banding: A Case and Technique Details Report

**DOI:** 10.3389/fcvm.2022.871818

**Published:** 2022-05-03

**Authors:** Yang Yu, Ruixuan Huang, Zheng Ding, Enyi Shi, Tianxiang Gu

**Affiliations:** Department of Cardiac Surgery, The First Hospital of China Medical University, Shenyang, China

**Keywords:** quadricuspid aortic valve (QAV), aortic valve replacement (AVR), aortic valve repair (AV repair), root banding, heart failiure

## Abstract

The quadricuspid aortic valve (QAV) is a rare congenital disease with a prevalence of 0. 013–0.043% of cardiac cases. Most patients with QAV are treated with aortic valve replacement. A Type B QAV with dilated ascending aorta of 47.9 mm; combined with severe regurgitation is reported here. In this case, considering the patient‘s cusps are flexible and reservable, the aortic root was reconstructed utilizing tricuspidization and annular banding technique, and dilated ascending aorta was replaced at the same time.

## Introduction

**Figure 1:**
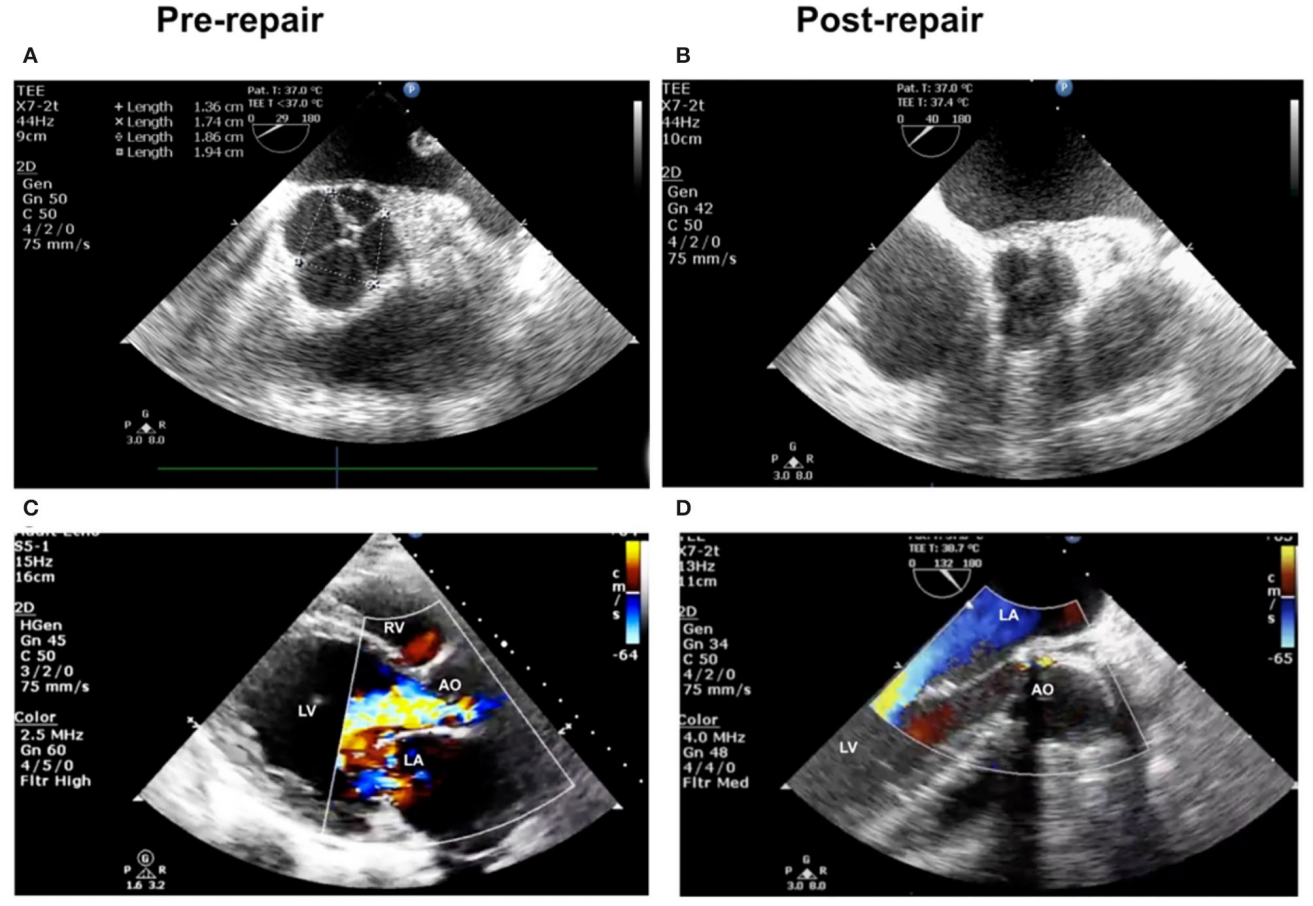
**(A)** Pre-operation: quadricuspid aortic valve (QAV). **(B)** Post-operation: QAV to tricuspidization aortic valve (TAV). **(C)** Transthoracic echocardiogram (TTE) demonstrated severe regurgitation before operation. **(D)** Post-operation image of transesophageal echocardiography (TEE) showed no regurgitation.

**Figure 2:**
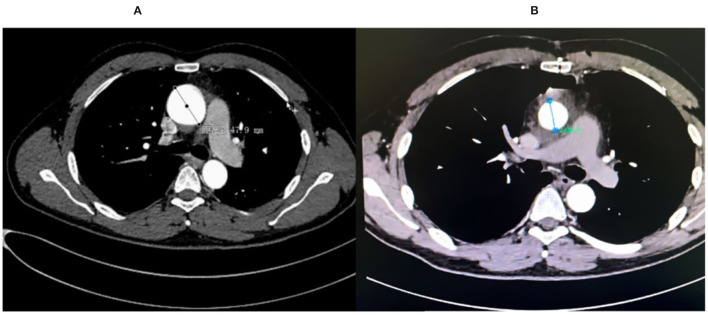
**(A)** The pre-op CT angiography (CTA) showed ascending aortic aneurysm of 47.9 mm. **(B)** The post-op CTA scan showed normal ascending aortic size.

The quadricuspid aortic valve (QAV) is a rare congenital disease that is prevalent in 0.013–0.043% of cardiac cases (1). Most patients with QAV are treated with aortic valve replacement (3). A patient with Type B QAV, a dilated ascending aorta of 47.9 mm, and severe regurgitation was reported here (2). In this case, considering that the patient's cusps were flexible and reservable, the aortic root was reconstructed utilizing tricuspidization and annular banding techniques as the dilated ascending aorta was replaced (Figures 1, 2).

## Case Presentation

A 45-year-old man suffered from a dysfunctional QAV (Hurwitz classification, type B) with severe aortic insufficiency (AI), decompensation heart failure, and intermittent atrial fibrillation. Transthoracic echocardiography (TTE) and transesophageal echocardiography (TEE) indicated a Type B QAV with a dilated ascending aorta of 47.9 mm (Figure 2A). TTE found that the left ventricular end-diastolic diameter was 77 mm and had a reduced ejection fraction of 39%. Modified “tricuspidization” of QAV, an innovative technique, and aortic valve annular banding were utilized to correct aortic valve insufficiency, and the ascending aorta was replaced at the same time.

**Figure 3:**
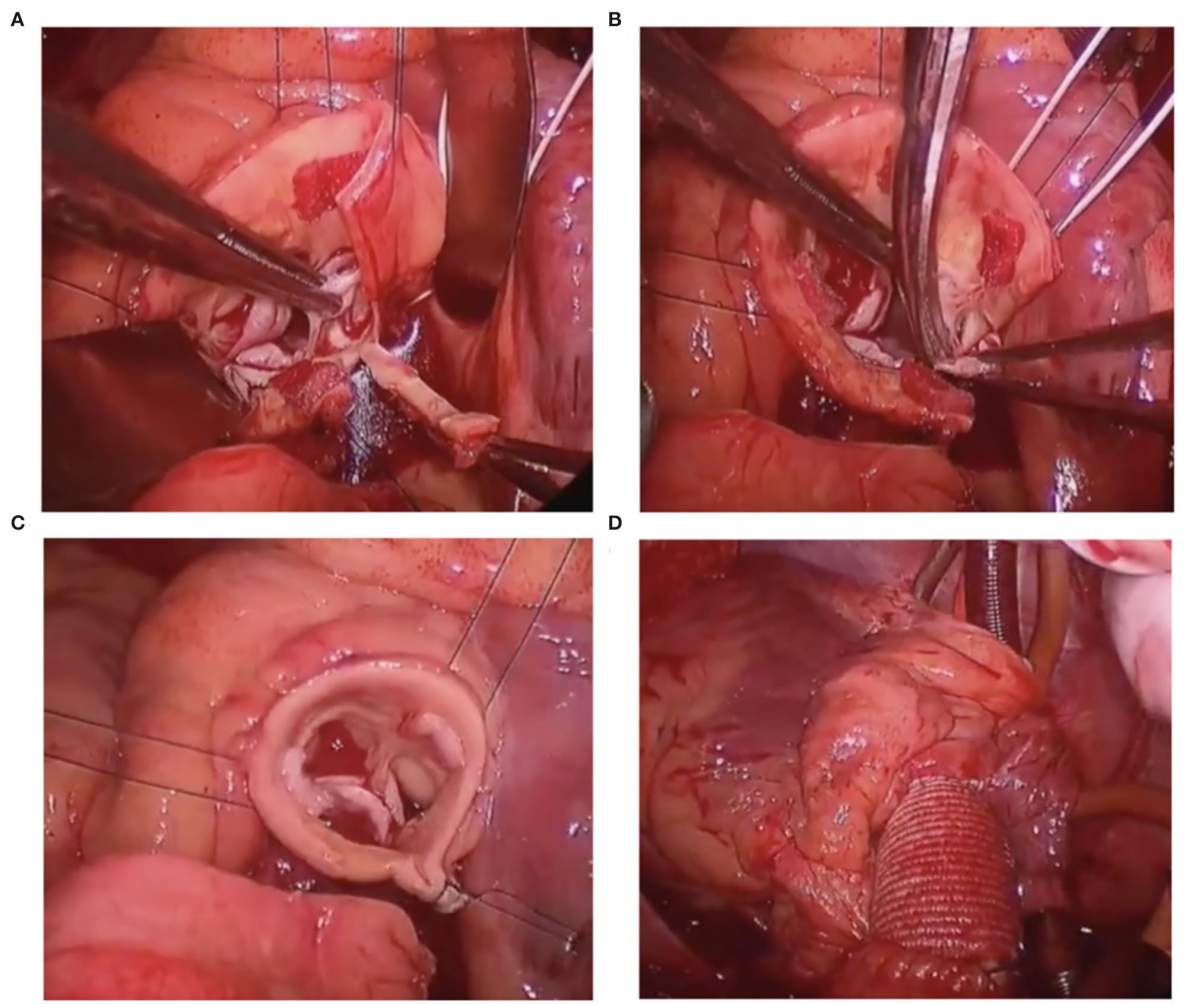
**(A)** Resect the aortic tissue from sinotubular junction (STJ) downwards to the sinus nadir of the smallest cusps. **(B)** Resect the smallest cusp. **(C)** Reconstruct the root by a horizontal running mattress suture. **(D)** Utilizing a 24-mm Dacron graft to replace the ascending aortic aneurysm.

Surgery was performed under moderate hypothermic cardiopulmonary bypass (CPB) support. After clamping, QAV was further confirmed by direct exploration. After cross-clamping, cardiac arrest was induced by antegrade cardioplegia solution perfusion and protected by continuous retrograde perfusion. Four interrupted U sutures were used to retract each commissure upward to assess the cusps after cutting open the ascending aorta transversely. QAV was asymmetrical, and there were three cusps of equal size and one smaller cusp. This smaller cusp was twisted and prolapsed, which contributed to the regurgitation. The aortic root was further dissected to the annular level, and two coronary orifices were identified. A continuous suture, using CV-0 sutures, was performed around the annulus. Thereafter, vertical resection of the smaller sinus and the cusp was performed. The aortic root was reconstructed by horizontal running mattress sutures, with 5-0 Prolene sutures, for “tricuspidization”. The CV-0 sutures were then tied with a 20-mm Hegar dilator into the left ventricular outflow tract (LVOT). Afterward, the aortic valve function, cusps position, and coaptation were carefully examined to ensure that there was no leakage or stenosis. Finally, a 24-mm Dacron graft was used to replace the ascending aorta (all the main surgical steps are shown in Figure 3).

Heartbeat was reinitiated, and there was no arrhythmia after unclamping. TEE showed that the residual three cusps functioned well without regurgitation or stenosis (Figures 1B,D). The patient recovered uneventfully and was discharged within 2 weeks. A short-term follow-up of 12 months showed no recurrence of AI, and the aortic aneurysm was resolved (Figure 2B). The left ventricle diameter decreased to 51 mm as the EF value increased to 64%. The patient did not experience significant complications or discomfort.

## Discussion

Dysfunctional QAV with aortic aneurysm is rare, and surgical repair to preserve the valve is challenging. Tricuspidization, a rare but efficient technique, was chosen to simultaneously repair aortic cusps and replace a dilated ascending aorta. A study of QAV by Dr. Pattersson and Dr. Svensson from Cleveland Clinic (4) indicated that 7 patients with QAV underwent surgical repair, 4 of whom underwent “tricuspidization”. According to Dr. Pattersson and Dr. Svensson, accessory cusps were resected but the sinus and related aortic root components were not resected. With their technique, residual cusps may have unequal pressure on blood flow. We modified “tricuspidization” by cutting off all the root tissue, including the cusp, sinus, and related STJ, to obtain better performance for blood flow based on our limited understanding. Annular banding was performed at the same time to avoid the recurrence of AI, which was attributed to dilation of the aortic annulus. Moreover, the Supplementary Materials show the surgical steps and techniques in detail (case presentation and video clips).

In some reports, QAVs are seen as abnormal septation of the truncus arteriosus or abnormal septation of one of the endocardial cushions (5). A biomechanical study of QAV has been reported for normal aortic valves (6), which means that QAVs have a high risk of forming aortic aneurysms due to congenital defects. In similar operations to correct QAV, transcatheter aortic valve implantation is used to cure QAV stenosis (7). A patient with a Type F QAV underwent surgery to remove the entire aortic root and repair the remaining cusps with its pericardial patch (8). Similarly, a patient with a Type A QAV underwent surgical repair to convert four aortic valves into two (9). The David procedure was applied to 2 QAV patients with dilated roots and AI in our center.

This technique prevents complications associated with anticoagulant use or reoperation. Therefore, valve function can be restored with better hemodynamic performance compared with that of prosthetic valves and other surgical techniques according to short-term follow-up. However, long-term follow-up is necessary to further evaluate the technique.

## Conclusion

The tricuspidization technique of asymmetrical QAV concomitant with aortic annulus banding can be implemented safely and effectively in well-selected patients with QAV. Larger cohort studies and long-term follow-up are needed to find a more durable and reliable technique to treat QAV.

## Data Availability Statement

The original contributions presented in the study are included in the article/Supplementary Material, further inquiries can be directed to the corresponding authors.

## Author Contributions

YY, ZD, and ES wrote the first draft of the manuscript. YY, ZD, ES, and TG wrote sections of the manuscript. All authors performed operation. All authors contributed to the article and approved the submitted version.

## Conflict of Interest

The authors declare that the research was conducted in the absence of any commercial or financial relationships that could be construed as a potential conflict of interest.

## Publisher's Note

All claims expressed in this article are solely those of the authors and do not necessarily represent those of their affiliated organizations, or those of the publisher, the editors and the reviewers. Any product that may be evaluated in this article, or claim that may be made by its manufacturer, is not guaranteed or endorsed by the publisher.
