# Supplementary figures and images for: Surgical Repair of a Quadricuspid Aortic Valve With Severe Regurgitation Utilizing “Tricuspidization” and Annular Banding: A Case and Technique Details Report
Source: Front Cardiovasc Med. 2022 May 3;9:871818. doi: 10.3389/fcvm.2022.871818 (PMC9110680; doi:10.3389/fcvm.2022.871818)

## Slide 1
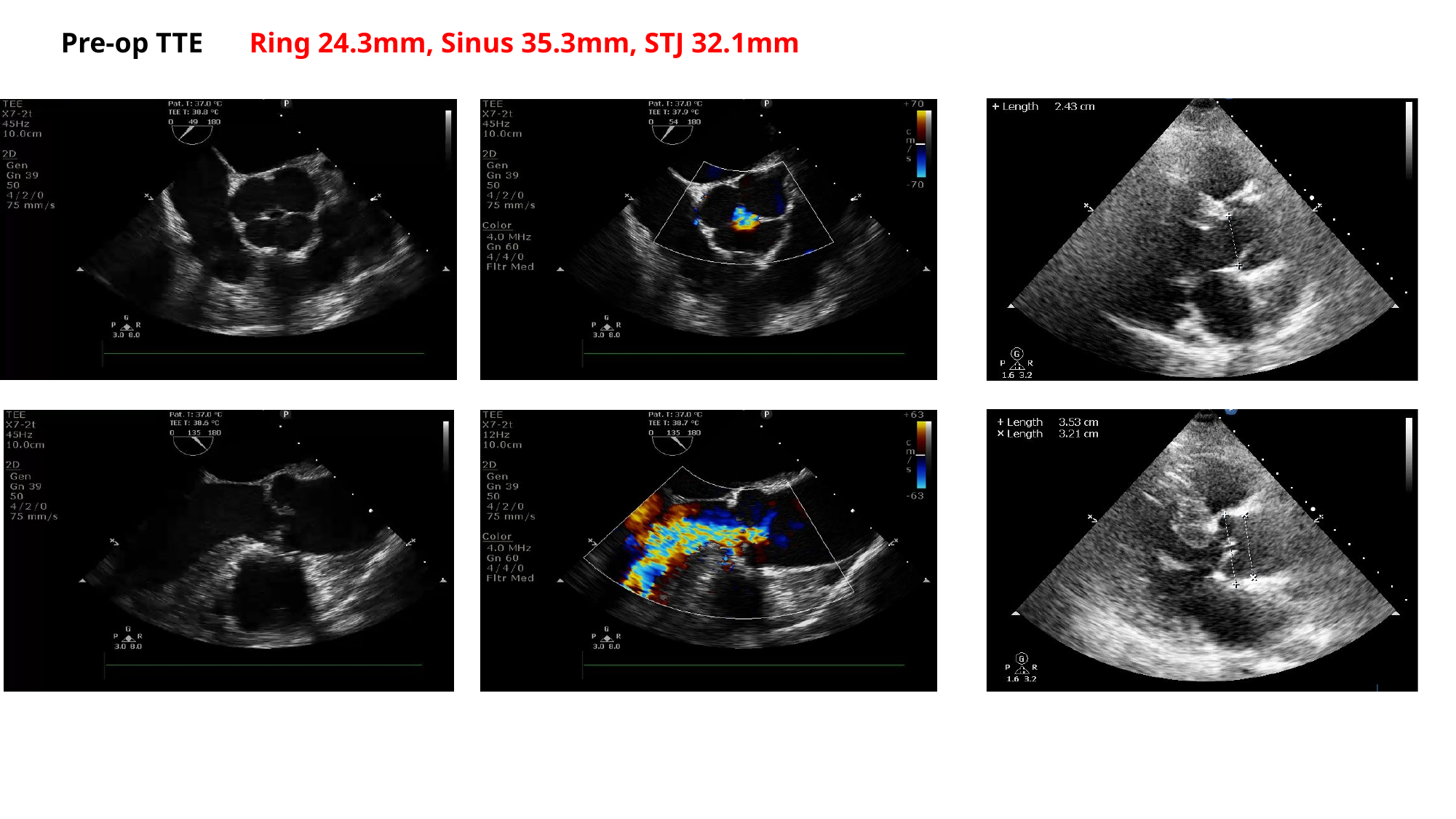

Ring 24.3mm, Sinus 35.3mm, STJ 32.1mm
Pre-op TTE

## Slide 2
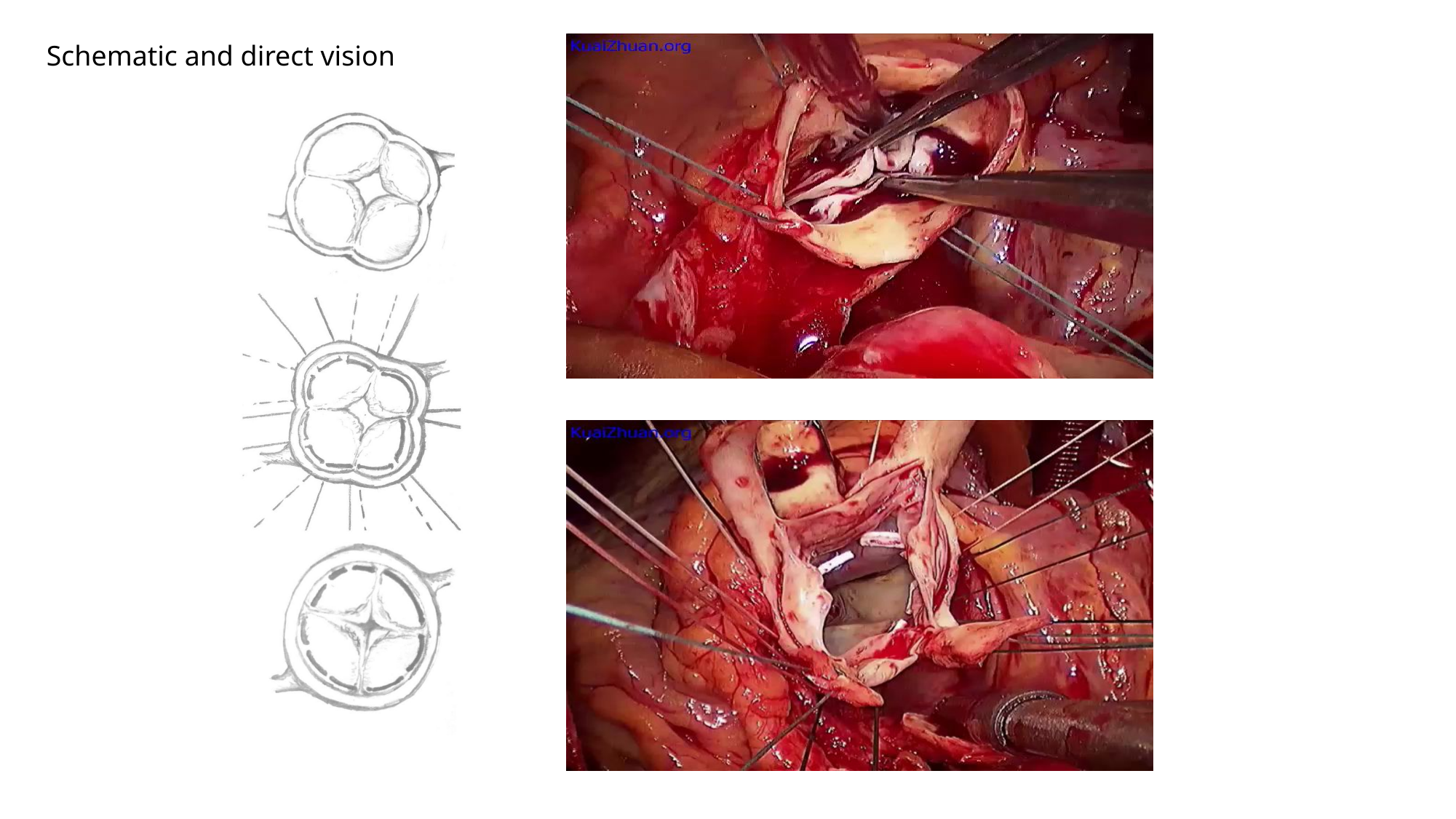

Schematic and direct vision

Supplement: Supplementary file 3 [file Presentation_1.PPTX]
